# Association of Smartphone Use Duration with Physical Fitness among University Students: Focus on Strength and Flexibility

**DOI:** 10.3390/ijerph19127386

**Published:** 2022-06-16

**Authors:** Wang Li, Yufei Cui, Qiang Gong, Zhihong Zhu

**Affiliations:** 1 Department of Physical Education, Huaiyin Institute of Technology, Huaian 223003, China; hzzh@hyit.edu.cn; 2 Department of Medicine and Science in Sports and Exercise, Tohoku University Graduate School of Medicine, Sendai 980-8575, Japan; qianggong0922@yahoo.co.jp

**Keywords:** smartphone, physical fitness, university students, cross-sectional study

## Abstract

High-frequency smartphone use leads to physical inactivity and may consequently cause the loss of physical fitness. Although the relationship between smartphone usage and physical fitness has been investigated, most of these studies focused on partial physical fitness, and the evaluation of the duration of smartphone use was insufficient. Therefore, this study aimed to investigate the relationship between the time period of using smart phones and physical fitness in Chinese university students. In this cross-sectional study, 8977 college students (5189 men and 3788 women) were enrolled. The assessment of smartphone usage was performed using a self-reported questionnaire. Physical fitness was measured in a sports facility, consisting of grip strength, standing long jump, and sit-and-reach test. The post adjustment relationship between smartphone use duration and physical fitness was examined by using the ANCOVA test. In the final adjusted model, an inverse association was observed between the smartphone use duration and grip strength in male and female participants (*p* = 0.003 and <0.001, respectively). The smartphone use duration was also negatively associated with standing long jump in both sexes (*p* = 0.003 for male and = 0.026 for female). In male participants, the longer the period of using smartphones, the lower their flexibility (*p* = 0.026). In conclusion, this study investigated the association between the duration of smartphone use and physical fitness. The results showed that longer durations of smartphone use were associated with weaker grip strength, a shorter standing long jump, and lower flexibility. Controlling smartphone-use duration might be beneficial for physical fitness among university students.

## 1. Introduction

Higher physical fitness is reported to be associated with many beneficial health outcomes, such as a lowered risk of cardiovascular disease [1], lower probability of prevalent and incident hypertension [2], and lower prevalence of type 2 diabetes [3]. It is also associated with better quality of life and cognitive function [4]. In addition, higher levels of physical fitness appear to reduce all-cause mortality [5]. Meanwhile, university or college is a critical period for young adults. University students face major changes in their lifestyle because they leave their parents’ home and live independently. The habits formed during this period may affect their later life. A study indicated that muscle strength is a powerful predictor of physical disability even 25 years later [6]. Thus, it is important to build higher physical fitness during young adulthood.

Meanwhile, smartphone ownership has become increasingly prevalent over the past decade. In 2018, there were more than 7.8 billion mobile phone users worldwide [7], and mobile phones have become an essential part of everyday life. Almost all university students in China own smartphones. Although smartphones are convenient to our lives, they also cause many problems. Previous studies found that adolescents use smartphones for more than 6.5 h everyday [8], and university students spend, on average, more than 3.5 h everyday using smartphones [9]. A study also indicates that students do not have sufficient control over their smartphone use [10]. In addition, a long duration of smartphone use may cause problems with people’s health. It has been proven that a high frequency of smartphone use is a risk factor for sleep disturbances and depressive symptoms [11] and is associated with perceived concentration difficulties [12], fatigue [12,13], headaches [14], and neck pain [15]. All these outcomes are risk factors for a decline in physical fitness. Thus, considering that the long-term use of smartphones reduces the time allocated for physical activity [16], this may also lead to a reduction in physical fitness. Although several studies investigated the relationship between smartphone usage and physical fitness, most of these studies focused on the relationship between smartphone usage, and hand function and grip strength [17,18,19]. To our knowledge, only one study has investigated the association between smartphone use and physical fitness, including strength, flexion, and agility in Spanish adolescents (12–18 years) [20]. However, no study has examined this association in university students, and the duration of smartphone use has not been sufficiently evaluated in previous studies. Thus, this study attempted to test whether smartphone use duration is associated with physical fitness among Chinese university students.

## 2. Materials and Methods

### 2.1. Participants

This study followed a cross-sectional design and was conducted in Huaian, East China. In total, 12,580 participants were invited from a university in Huaian City in 2018. This study was based on the annual physical health examination of university students. We conducted a questionnaire survey after the students’ physical health examinations. Participants joined the study voluntarily, and written consent was obtained from all students before the survey. This study was approved by the institutional ethics committee of the Huaiyin Institute of Technology (approval number: 2018RL-401). We excluded 3603 participants owing to missing data on smartphone use (*n* = 533) and physical fitness (*n* = 2266) or unavailable data for other variables. In addition, students who had physical disabilities, cardiovascular diseases, respiratory diseases and a special reason or circumstance that prevented them from participation were excluded. Thus, 8977 participants (5189 men and 3788 women) were included in this study.

### 2.2. Assessment of Smartphone Use

The duration of smartphone use was evaluated using the following question: “for how long do you usually use your smartphone in a day”. The total duration of smartphone use was calculated from the students’ responses. Then, the reported daily durations of smartphone use were divided into the following tertiles: short (≤240 min), medium (241–360 min), and long (>360 min).

### 2.3. Assessment of Physical Fitness

All measurements of physical fitness were based on the National Student Physical Health Examination (revised in 2014) [21], which was developed for monitoring and evaluating the physical fitness changes in Chinese students (from primary school to university). We conducted the following physical fitness tests by using the criterion for university students.

#### 2.3.1. Grip Strength (Upper Body Strength)

Grip strength was measured using a hand-held grip dynamometer (WCS-10000 Wanqing Electron Co., Shanghai, China). The grip strength of each hand was accurately measured twice. Participants were in a standing position and were encouraged to squeeze the handle as hard as possible with their arms straight on the side of their body. The maximum force (kg) applied was used for the analysis.

#### 2.3.2. Standing Long Jump (Lower Body Strength)

A standing long jump was performed on a track and field ground. The participants were asked to stand behind a line marked on the ground. A two-foot take-off and landing was used, with swinging of the arms and bending of the knees to provide a forward drive. The participants attempted to jump as far as possible and land on both feet without falling backward. The measurement was taken from the take-off line to the nearest point of contact on landing (back of the heels). Two attempts were required, and the longest distance (cm) was considered for the analysis.

#### 2.3.3. Sit-and-Reach Test (Flexibility)

In the sit-and-reach test, a professional device (JH-1441 Jihao Electron Co., Changzhou, China) was used. Participants were asked to sit on the floor with their legs stretched out straight ahead, and shoes were removed. The feet were placed flat against the board. Both knees were locked and pressed flat on the floor. With the palms facing downward, and the hands on top of each other or side by side, after ensuring that the hands remained at the same level, the participants reached forward slowly along the measuring line as far as possible. The distance (cm) covered during each participant’s reach was recorded.

### 2.4. Confounding Factors

We considered the following variables as potential confounding factors: sex (male and female), grade (freshmen, sophomore, junior), living expenses [low (≤1000 yuan/month), medium (1001–1500 yuan/month), and high (>1500 yuan/month)], race (Han and minority races), living status (dormitory and others), sleep duration (7–8 h of sleep duration, among others), smoking status (smoker and non-smoker), alcohol drinking status (non-drinker, drinking 1–2 times/week, and drinking >2 times/week), body mass index (BMI) in weight/height^2^ (kg/m^2^) (continuous variable). The daily physical activity (PA) was evaluated using the International Physical Activity Questionnaire (IPAQ) [22]. The daily total PA was calculated as follows: metabolic equivalents [METs] × h/week. The PA was divided into tertiles (low, medium, and high). Depressive symptoms were assessed using the Zung Self-Rating Depression Scale (SDS) [23]. Students with an SDS score ≥ 45 were considered depressed [24].

### 2.5. Statistical Analysis

Participant characteristics according to the duration of smartphone-use categories were compared using a t-test or analysis of variance (ANOVA) for continuous variables and χ^2^ test for categorical variables. Results were expressed as the mean (95% CI) for continuous variables and percentage for categorical variables. Differences in physical fitness (dependent variable) according to the duration of smartphone use (independent variable) were examined using the analysis of covariance (ANCOVA) test after multivariate adjustment. Grade, BMI, and race were adjusted for in Model 1. The items in Model 1 and living expenses, PA, living status, smoking and drinking status, depressive symptoms, and sleep duration were controlled for in Model 2. Results are expressed as means with 95% confidence intervals (95% CIs). All analyses were performed using the Statistical Package for Social Science (SPSS) (version 24.0; IBM Corporation, Armonk, NY, USA) for Windows^®^. Statistical significance was set at *p* < 0.05.

## 3. Results

Table 1 shows the characteristics of participants by gender. BMI, sit-and-reach test scores and the duration of smartphone use in male participants was lower than that of female participants. Male participants also had longer standing long jump distances and higher grip strengths. The proportions of low living expenses, drinking 1–2 times/week, and drinking >2 times/week were higher in male participants than in female participants. Female participants had a higher proportion of medium and high living expenses, minority race, nonsmokers, non-drinkers, and people with a 7–8 h/day sleep duration.

Table 2 shows the characteristics of male and female participants according to the duration of smartphone-use categories. In male participants, with the increasing duration of smartphone use, the proportion of freshmen, people with low living expenses, nonsmokers, non-drinkers, and people who sleep for 7–8 h a day decreased significantly (trend *p* < 0.001, 0.017, 0.001, 0.007, and <0.001, respectively); conversely, the proportion of sophomores, people who drink 1–2 times/week, and those who have depressive symptoms increased significantly (trend *p* < 0.001, 0.010, and <0.001, respectively). Among female participants, with ana increasing duration of smartphone use, the proportion of freshmen, people with low living expenses, nonsmokers, non-drinkers, and people who sleep for 7–8 h a day decreased significantly (trend *p* < 0.001, <0.001, 0.009, 0.007, and <0.001, respectively), while the proportion of junior, medium, and high living expenses, drinking >2 times/week, and those who had depressive symptoms increased significantly (trend *p* < 0.001, 0.021, <0.001, <0.001, and <0.001, respectively).

The post-adjusted association between smartphone-use duration and physical fitness in male participants is provided in Table 3. In model 1, grip strength (kg) across categories of smartphone-use duration was 39.9 (39.6, 40.2) for short duration, 39.7 (39.3, 40.0) for medium duration, and 39.3 (39.0, 39.7) for long duration. Standing long jump (cm) across categories of smartphone-use duration were 230.4 (229.6, 231.1) for short duration, 230.1 (229.2, 231.0) for medium duration, and 228.9 (227.9, 229.8) for long duration. Sit-and-reach distance (cm) across categories of smartphone-use duration were 11.6 (11.3, 11.9) for short duration, 11.2 (10.9, 11.5) for medium duration, and 11.1 (10.8, 11.5) for long duration. These significant associations did not change in model 2.

The adjusted association between female participants’ smartphone-use duration and physical fitness is presented in Table 4. Inverse associations were also found between duration of smartphone use and grip strength and standing long jump in females. In model 1, grip strength (kg) across categories of smartphone-use duration was 23.2 (23.0, 23.5) for short duration, 22.8 (22.6, 23.1) for medium duration, and 22.5 (22.2, 22.7) for long duration. Standing long jump (cm) across categories of smartphone-use duration were 171.6 (170.8, 172.4) for short duration, 171.2 (170.4, 172.0) for medium duration, and 170.3 (169.4, 171.1) for long duration. These significant associations did not change in Model 2. However, no significant association was found between the duration of smartphone use and the sit-and-reach test scores in female participants.

## 4. Discussion

This was a cross-sectional study. We verified the association between smartphone-use duration and physical fitness among Chinese university students. According to our findings, longer durations of smartphone use were associated with lower grip strength (upper body strength), shorter standing long jump (lower body strength), and shorter sit and reach (flexibility). These associations were independent of confounding factors such as sex, BMI, grade, race, health status, and lifestyle. To the best of our knowledge, this is the first study to investigate the association between the duration of smartphone use and physical fitness among Chinese university students. Our findings strengthen the existing evidence on mobile phone use, physical fitness, and student health.

In the present study, a significant difference in smartphone use was found between sexes; female students used smartphones (6.1 h/day) for longer than male students (5.5 h/day). These results were similar to those of a study carried out in Jordan, which revealed that the average daily smartphone use of a university student was 6.9 h for females and 5.6 h for males [25]. This is also consistent with another study that showed higher smartphone addiction values in female university students [26]. However, our findings are different from those of studies carried out in Turkey [27], Saudi Arabia [19], and Canada [9], which indicated that the average daily duration of smartphone use of university students was 7.85 h, 7.8 h, and 3.5 h, respectively. Students from different countries may have different smartphone-use habits, which may lead to differences in the findings of these previous studies. However, compared to these studies, the smartphone-use duration in our study was relatively short.

Some studies revealed the relationship between smartphone use and grip strength. A study of 102 university students investigated the flexor pollicis longus tendon and median nerve in smartphone users using ultrasonography to assess the effects of smartphone addiction on the clinical and functional status of the hands. The results of the study showed that smartphone overuse decreased pinch strength and hand function [17]. The aim and design of that study were not consistent with those of our study; however, the results support our findings given that a decrease in hand function is a risk factor for poor grip strength. Another study of children aged 9–15 years investigated the interaction effects between smartphone usage level and hand dominance on pinch strength, handgrip, and hand performance. This randomized trial study revealed that hand grip and pinch strength were reduced in high-frequency smartphone users, especially in the dominant hand [18]. The finding of that study is similar to our finding. However, that study considered the difference between the dominant and non-dominant hand. In our study, we used only the grip strength of the stronger hand. In addition, a study conducted in Saudi Arabia aimed to investigate the association between smartphone-use duration and handgrip strength. The results showed prolonged smartphone use to be associated with weaker handgrip strength in 100 young males [19]. The study had the same objectives and results as our study. In general, the results of the aforementioned studies are consistent with our present findings. However, elements of physical fitness other than grip strength were not examined in these previous studies. On the other hand, a study carried out in Spain investigated the relationship between smartphone use and muscle strength, flexion, and agility in 501 high school students [20]. They found that the high use of mobile phones was associated with worse results in physical fitness tests. In this study, six items were used to define physical fitness; two of them (standing long jump and trunk flexion test) were also used in our study. However, the assessment of smartphone use and partial physical fitness differed from those in our study. All of these studies, including ours, indicated that a high frequency of smartphone usage was related to lower physical fitness. Furthermore, our findings extend this relationship to university students.

Although the mechanism of the association between the duration of smartphone usage and physical fitness is not clear, there are several plausible explanations as to why the duration of smartphone use is associated with physical fitness. First, when a smartphone is constantly used without regular rest, or a poor posture is maintained over a long period of time, musculoskeletal fatigue and pain can develop as a result. Second, during long periods of smartphone use, most body muscles do not perform sufficient physical activity to promote physical fitness. Third, a study indicated that smartphone addiction results in an increase in fat mass and a decrease in muscle mass [28]. Low muscle mass is strongly associated with low muscle strength [29]; thus, this might indirectly cause the loss in overall physical fitness. Fourth, cognitive function may be an important mediator between smartphone use and physical fitness because excessive smartphone use can cause cognitive function impairment [30], and better cognitive function is associated with better physical fitness [31]. Finally, it has been reported that excessive smartphone use is associated with poor sleep quality among Chinese college students [32]. Poor sleep quality is also a risk factor for the loss of physical fitness. All these factors demonstrate why longer smartphone use is associated with lower physical fitness.

Although the study sample size was large, and many confounding factors were adjusted for in the analysis, this study has several limitations. First, we collected self-reported data on lifestyle, depression, and sleep. Although some scales such as the IPAQ, SDS, and PSQI were validated, there might still be recall bias. Second, this was a cross-sectional study. Thus, we could not establish causality. Third, the current results may not be representative of all Chinese university students because the participants were only from Huaian City, China. Fourth, this study was conducted with the help of up to 30 instructors. Although instructors were trained before the physical health examination to unify the measurement skills, there may also be measurement errors between them. Fifth, we only assessed smartphone use with a question regarding the duration of smartphone use; other smartphone or computer-use habits such as problematic use or addiction, which may be mediators for the association between smartphone use and physical fitness, were not evaluated in the study. Finally, while we collected data on the basic confounding factors, we cannot exclude the possibility that physical fitness is affected by other factors associated with smartphone use, such as diet or academic pressure.

## 5. Conclusions

The current study revealed that a longer smartphone-use duration was related to a lower level of physical fitness (strength and flexibility) among Chinese university students. Therefore, these findings imply that controlling daily smartphone-use duration is recommended to prevent the loss of physical fitness in university students. Our study provides solid evidence for the physical health of young adults and its implications in the field of preventive medicine and health education. Future well-designed prospective studies or randomized studies are required to confirm these findings and establish causality.

## Figures and Tables

**Table 1:** Basic characteristics of male and female participants.

	Male	Female	*p* Value ^a^
*n*	5189	3788	
BMI (kg/m^2^) ^b^	22.0 (21.9, 22.1) ^c^	20.4 (20.3, 20.5)	<0.001
Grade (%)			
Freshman	28.4	29.2	0.409
Sophomore	37.5	37.1	0.724
Junior	34.1	33.7	0.685
Living expenses (%)			
Low	38.0	34.2	<0.001
Medium	52.3	54.7	0.023
High	9.7	11.1	0.027
Minority race (%)	4.0	5.9	<0.001
Living status (Dormitory; %)	99.1	99.3	0.406
Nonsmoker (%)	91.5	99.5	<0.001
Drinking status (%)			
Nondrinker	68.8	95.4	<0.001
Drink 1–2 times/week	28.9	4.2	<0.001
Drink >2 times/week	2.3	0.3	<0.001
PA (METs hour/week)	51.4 (50.2, 52.7)	51.2 (49.7, 52.7)	0.809
Sleep duration (7–8 h/day; %)	54.5	60.1	<0.001
Depressive symptoms (%)	11.5	12.3	0.290
Duration of smartphone use (Minutes/day)	327.8 (322.9, 332.6)	367.0 (361.3, 372.7)	<0.001
Standing long jump (cm)	229.9 (229.4, 230.4)	171.1 (170.5, 171.6)	<0.001
Grip strength (kg)	39.7 (39.5, 39.8)	22.8 (22.6, 23.0)	<0.001
Sit and reach test (cm)	11.3 (11.2, 11.5)	16.7 (16.5, 16.9)	<0.001

^a^ Obtained using ANOVA for continuous variables and x^2^ test for proportional variables. ^b^ BMI: body mass index. PA: physical activity. ^c^ Mean; 95% CI in parentheses (all such values).

**Table 2:** Characteristics of male and female participants according to duration of smartphone use.

	Duration of Smartphone Use			Trend p ^a^
	Short	Medium	Long	
Male participants				
n	2213	1580	1396	
BMI (kg/m^2^) ^b^	22.0 (21.8, 22.1) ^c^	22.0 (21.8, 22.1)	22.0 (21.8, 22.1)	0.927
Grade (%)				
Freshman	31.5	28.7	23.1	<0.001
Sophomore	35.2	36.2	42.7	<0.001
Junior	33.3	35.1	34.2	0.485
Living expenses (%)				
Low	40.0	36.9	36.2	0.017
Medium	51	53	53.4	0.127
High	9	10.1	10.3	0.184
Minority race (%)	4.3	4.1	3.2	0.107
Living status (Dormitory; %)	99.2	99.1	98.9	0.343
Nonsmoker (%)	92.8	91.3	89.7	0.001
Drinking status (%)				
Nondrinker	71	67.1	67.0	0.007
Drink 1–2 times/week	26.9	30.2	30.7	0.010
Drink >2 times/week	2.1	2.6	2.3	0.581
PA (METs hour/week)	49.5 (47.6, 51.5)	49.6 (47.4, 51.9)	56.5 (54.1, 58.9)	<0.001
Sleep duration (7–8 h/day; %)	58.2	55.8	47.1	<0.001
Depressive symptoms (%)	9.2	12.5	14.2	<0.001
Female participants				
n	1297	1286	1205	
BMI (kg/m^2^)	20.5 (20.3, 20.6)	20.5 (20.3, 20.6)	20.4 (20.2, 20.5)	0.386
Grade (%)				
Freshman	35.5	27.9	23.8	<0.001
Sophomore	36.9	38.9	35.5	0.512
Junior	27.6	33.2	40.7	<0.001
Living expenses (%)				
Low	38.6	34.2	29.3	<0.001
Medium	52.4	54.8	57	0.021
High	8.9	11	13.7	<0.001
Minority race (%)	6.7	5.1	5.7	0.283
Living status (Dormitory; %)	99.5	99.4	99	0.179
Nonsmoker (%)	99.7	99.8	98.9	0.009
Drinking status (%)				
Nondrinker	96.5	95.6	94.2	0.007
Drink 1–2 times/week	3.5	4.4	4.9	0.074
Drink >2 times/week	0.1	0.1	0.9	<0.001
PA (METs hour/week)	48.0 (45.4, 50.5)	50.6 (48.1, 53.3)	55.2 (52.5, 57.8)	<0.001
Sleep duration (7–8 h/day; %)	62.7	62.1	55.1	<0.001
Depressive symptoms (%)	8.9	12.1	16	<0.001

^a^ Obtained using ANOVA for continuous variables and x^2^ test for proportional variables. ^b^ BMI: body mass index. PA: physical activity. ^c^ Mean; 95% CI in parentheses (all such values).

**Table 3:** Adjusted relationship between smartphone-use duration and physical fitness in male participants.

	Duration of Smartphone Use			
	Short	Medium	Long	Trend p ^a^
n.	2213	1580	1396	
Grip strength (kg)				
Crude	39.8 (39.5, 40.1) ^b^	39.7 (39.3, 40.0)	39.4 (39.0, 39.8)	0.075
Model 1 ^c^	39.9 (39.6, 40.2)	39.7 (39.3, 40.0)	39.3 (39.0, 39.7)	0.013
Model 2 ^d^	39.9 (39.7, 40.2)	39.7 (39.4, 40.0)	39.2 (38.9, 39.6)	0.003
Standing long jump (cm)				
Crude	230.3 (229.5, 231.1)	230.1 (229.2, 231.0)	229.0 (228.0, 230.0)	0.044
Model 1 ^c^	230.4 (229.6, 231.1)	230.1 (229.2, 231.0)	228.9 (227.9, 229.8)	0.014
Model 2 ^d^	230.5 (229.7, 231.2)	230.2 (229.3, 231.0)	228.7 (227.7, 229.6)	0.003
Sit and reach test (cm)				
Crude	11.6 (11.4, 11.9)	11.2 (10.9, 11.5)	11.1 (10.8, 11.4)	0.014
Model 1 ^c^	11.6 (11.3, 11.9)	11.2 (10.9, 11.5)	11.1 (10.8, 11.5)	0.025
Model 2 ^d^	11.6 (11.3, 11.9)	11.2 (10.9, 11.5)	11.1 (10.8, 11.4)	0.026

^a^ Obtained using ANCOVA. ^b^ Values were expressed as means (95% CI). ^c^ Adjustment for grade, body mass index and race. ^d^ Adjustment for items in Model 1 plus living expenses, living status, physical activity, smoking and drinking habits, depressive symptoms and sleep duration.

**Table 4:** Adjusted relationship between duration of smartphone use and physical fitness in female participants.

	Duration of Smartphone Use			
	Short	Medium	Long	Trend p ^a^
n.	1297	1286	1205	
Grip strength (kg)				
Crude	23.2 (23.0, 23.5) ^b^	22.8 (22.6, 23.1)	22.5 (22.2, 22.7)	<0.001
Model 1 ^c^	23.2 (23.0, 23.5)	22.8 (22.6, 23.1)	22.5 (22.2, 22.7)	<0.001
Model 2 ^d^	23.2 (22.9, 23.4)	22.8 (22.6, 23.1)	22.5 (22.3, 22.8)	<0.001
Standing long jump (cm)				
Crude	171.3 (170.5, 172.1)	171.2 (170.3, 172.0)	170.7 (169.8, 171.5)	0.268
Model 1 ^c^	171.6 (170.8, 172.4)	171.2 (170.4, 172.0)	170.3 (169.4, 171.1)	0.023
Model 2 ^d^	171.6 (170.8, 172.4)	171.2 (170.4, 172.0)	170.3 (169.4, 171.1)	0.026
Sit and reach test (cm)				
Crude	16.8 (16.5, 17.1)	16.4 (16.1, 16.7)	16.8 (16.5, 17.1)	0.886
Model 1 ^c^	16.8 (16.5, 17.1)	16.4 (16.1, 16.7)	16.7 (16.4, 17.1)	0.783
Model 2 ^d^	16.8 (16.5, 17.1)	16.4 (16.1, 16.7)	16.8 (16.5, 17.1)	0.990

^a^ Obtained using ANCOVA. ^b^ Values were expressed as means (95% CI). ^c^ Adjustment for age, body mass index and race. ^d^ Adjustment for items in Model 1 plus living expenses, living status, physical activity, smoking and drinking habits, depressive symptoms and sleep duration.

## Data Availability

The datasets used and analyzed during the current study are available from the corresponding author on reasonable request.

## References

[1] Kaminsky L.A., Arena R., Ellingsen O., Harber M.P., Myers J., Ozemek C., Ross R. (2019). Cardiorespiratory fitness and cardiovascular disease—The past, present, and future. Prog. Cardiovasc. Dis..

[2] Juraschek S.P., Blaha M.J., Whelton S.P., Blumenthal R., Jones S.R., Keteyian S.J., Schairer J., Brawner C.A., Al-Mallah M.H. (2014). Physical fitness and hypertension in a population at risk for cardiovascular disease: The Henry Ford ExercIse Testing (FIT) Project. J. Am. Heart Assoc..

[3] Momma H., Sawada S.S., Kato K., Gando Y., Kawakami R., Miyachi M., Huang C., Nagatomi R., Tashiro M., Ishizawa M. (2019). Physical Fitness Tests and Type 2 Diabetes Among Japanese: A Longitudinal Study From the Niigata Wellness Study. J. Epidemiol..

[4] Daimiel L., Martinez-Gonzalez M.A., Corella D., Salas-Salvado J., Schroder H., Vioque J., Romaguera D., Martinez J.A., Warnberg J., Lopez-Miranda J. (2020). Physical fitness and physical activity association with cognitive function and quality of life: Baseline cross-sectional analysis of the PREDIMED-Plus trial. Sci. Rep..

[5] Blair S.N., Kohl H.W., Paffenbarger R.S., Clark D.G., Cooper K.H., Gibbons L.W. (1989). Physical fitness and all-cause mortality. A prospective study of healthy men and women. JAMA.

[6] Rantanen T., Guralnik J.M., Foley D., Masaki K., Leveille S., Curb J.D., White L. (1999). Midlife hand grip strength as a predictor of old age disability. JAMA.

[7] Gunnell K.E., Flament M.F., Buchholz A., Henderson K.A., Obeid N., Schubert N., Goldfield G.S. (2016). Examining the bidirectional relationship between physical activity, screen time, and symptoms of anxiety and depression over time during adolescence. Prev. Med..

[8] Odgers C. (2018). Smartphones are bad for some teens, not all. Nature.

[9] Berolo S., Wells R.P., Amick B.C. (2011). Musculoskeletal symptoms among mobile hand-held device users and their relationship to device use: A preliminary study in a Canadian university population. Appl. Ergon..

[10] Lee J., Cho B., Kim Y., Noh J. (2015). Smartphone addiction in university students and its implication for learning. Emerging Issues in Smart Learning.

[11] Thomee S., Harenstam A., Hagberg M. (2011). Mobile phone use and stress, sleep disturbances, and symptoms of depression among young adults—A prospective cohort study. BMC Public Health.

[12] Johansson A., Nordin S., Heiden M., Sandstrom M. (2010). Symptoms, personality traits, and stress in people with mobile phone-related symptoms and electromagnetic hypersensitivity. J. Psychosom. Res..

[13] Kim S.Y., Koo S.J. (2016). Effect of duration of smartphone use on muscle fatigue and pain caused by forward head posture in adults. J. Phys. Ther. Sci..

[14] Cho Y.M., Lim H.J., Jang H., Kim K., Choi J.W., Shin C., Lee S.K., Kwon J.H., Kim N. (2016). A cross-sectional study of the association between mobile phone use and symptoms of ill health. Environ. Health Toxicol..

[15] Lee S., Kang H., Shin G. (2015). Head flexion angle while using a smartphone. Ergonomics.

[16] Lajunen H.R., Keski-Rahkonen A., Pulkkinen L., Rose R.J., Rissanen A., Kaprio J. (2007). Are computer and cell phone use associated with body mass index and overweight? A population study among twin adolescents. BMC Public Health.

[17] Inal E.E., DemIrc I.K., CetInturk A., Akgonul M., Savas S. (2015). Effects of smartphone overuse on hand function, pinch strength, and the median nerve. Muscle Nerve.

[18] Radwan N.L., Ibrahim M.M., Mahmoud W.S.E. (2020). Evaluating hand performance and strength in children with high rates of smartphone usage: An observational study. J. Phys. Ther. Sci..

[19] Osailan A. (2021). The relationship between smartphone usage duration (using smartphone’s ability to monitor screen time) with hand-grip and pinch-grip strength among young people: An observational study. BMC Musculoskelet. Disord..

[20] Bravo-Sanchez A., Moran-Garcia J., Abian P., Abian-Vicen J. (2021). Association of the Use of the Mobile Phone with Physical Fitness and Academic Performance: A Cross-Sectional Study. Int. J. Environ. Res. Public Health.

[21] Ministry of Education of the People’s Republic of China (2014). Notification of National Student Physical Health Standard.

[22] Craig C.L., Marshall A.L., Sjostrom M., Bauman A.E., Booth M.L., Ainsworth B.E., Pratt M., Ekelund U., Yngve A., Sallis J.F. (2003). International physical activity questionnaire: 12-country reliability and validity. Med. Sci. Sports Exerc..

[23] Zung W.W. (1965). A Self-Rating Depression Scale. Arch. Gen. Psychiatry.

[24] Cui Y., Huang C., Momma H., Ren Z., Sugiyama S., Guan L., Niu K., Nagatomi R. (2017). Consumption of low-fat dairy, but not whole-fat dairy, is inversely associated with depressive symptoms in Japanese adults. Soc. Psychiatry Psychiatr. Epidemiol..

[25] Al-Hadidi F., Bsisu I., AlRyalat S.A., Al-Zu’bi B., Bsisu R., Hamdan M., Kanaan T., Yasin M., Samarah O. (2019). Association between mobile phone use and neck pain in university students: A cross-sectional study using numeric rating scale for evaluation of neck pain. PLoS ONE.

[26] Del Rio M.I.P., Lazaro S.M., Del Barco B.L., Castano E.F. (2017). Mobile Abuse in University Students and profiles of victimization and aggression. Adicciones.

[27] Kaya F., Dastan N.B., Durar E. (2020). Smart phone usage, sleep quality and depression in university students. Int. J. Soc. Psychiatry.

[28] Kim S.E., Kim J.W., Jee Y.S. (2015). Relationship between smartphone addiction and physical activity in Chinese international students in Korea. J. Behav. Addict..

[29] Kim S.E., Hong J., Cha J.Y., Park J.M., Eun D., Yoo J., Jee Y.S. (2016). Relative appendicular skeletal muscle mass is associated with isokinetic muscle strength and balance in healthy collegiate men. J. Sports Sci..

[30] Al-Khlaiwi T.M., Habib S.S., Meo S.A., Alqhtani M.S., Ogailan A.A. (2020). The association of smart mobile phone usage with cognitive function impairment in Saudi adult population. Pak. J. Med. Sci..

[31] Kang S.J., Kim B.H., Lee H., Wang J. (2022). Association among cognitive function, physical fitness, and health status in older women. J. Exerc. Rehabil..

[32] Huang Q., Li Y., Huang S., Qi J., Shao T., Chen X., Liao Z., Lin S., Zhang X., Cai Y. (2020). Smartphone Use and Sleep Quality in Chinese College Students: A Preliminary Study. Front. Psychiatry.

